# Association between *RGS4* gene polymorphisms and schizophrenia: A protocol for systematic review and meta-analysis

**DOI:** 10.1097/MD.0000000000027607

**Published:** 2021-11-05

**Authors:** Feng-Ling Xu, Jun Yao, Bao-Jie Wang

**Affiliations:** School of Forensic Medicine, China Medical University, Shenyang, China.

**Keywords:** meta-analysis, pooled analysis, *RGS4*, schizophrenia

## Abstract

**Background:**

Schizophrenia is a complex brain disorder, the pathogenesis of which remains unclear. Regulator of G-protein signaling 4 is regarded as a candidate gene for schizophrenia risk. The association between the regulator of G-protein signaling 4 gene and the risk of schizophrenia is complicated and controversial, thus, an updated meta-analysis is needed.

**Methods:**

A search strategy using Medical Subject Headings was developed in English (PubMed, SZGene) and Chinese (CNKI, Wanfang, and Weipu) databases. Inclusion and exclusion criteria were used to screen for eligible studies. Parameters, such as *P* value of Hardy–Weinberg equilibrium, odds ratios, 95% confidence intervals, *P* values of association, heterogeneity (*P*_*h*_), and publication bias, were analyzed by the Stata software using a random effects model. Subgroup analyses were performed to detect heterogeneity.

**Results:**

There were 15 articles regarding rs10917670 (8046 cases and 8837 controls), 16 regarding rs951436 (8990 cases and 10,568 controls), 15 regarding rs951439 (7995 cases and 8646 controls), 15 regarding rs2661319 (8320 cases and 9440 controls), and 4 regarding rs10759 (2752 cases and 2866 controls). The frequencies of rs10917670 and rs951439 were not significantly different between the case and control groups (*P* > .05). As shown by the East Asian and hospital-based subgroup analyses, the genotype TT of rs951436 might be related to the risk of schizophrenia. The genotypes CC + CT of rs2661319 and CC + CA of rs10759 were statistically different between the 2 groups, and the East Asian population contributed to these differences.

**Conclusion:**

The genotypes CC + CT of rs2661319 and CC + CA of rs10759 might be associated with the risk of schizophrenia.

## 1 Introduction

Schizophrenia is a complex brain disorder, the pathogenesis of which remains unclear.^[1]^ It has been shown that schizophrenia is caused by both genetic and environmental factors,^[2]^ and genetic factors play an important role to the etiology of schizophrenia.^[3,4]^ Regulator of G-protein signaling proteins control the duration and timing of intracellular signaling of many G-protein coupled receptors. The major mechanism by which regulator of G-protein signaling proteins negatively regulate G proteins is via their GTPase accelerating activity.^[5]^ Regulator of G-protein signaling 4 (RGS4) is known to play a fundamental role in neurotransmission and neuronal differentiation, in addition to axonogenesis during embryogenesis.^[6]^ RGS4 regulation of G-protein activity, may inhibit the interaction between neurotransmitters and their receptors, leading to dysfunction of glutamatergic neurotransmission,^[7]^ which is classically related to the etiology of psychotic disorders.^[8]^ Schwarz et al^[6]^ suggested that the *RGS4* gene, localized to chromosome 1q23, might be an important part of a larger biological system contributing to schizophrenia risk. Mirnics et al^[9]^ showed that *RGS4* expression was down regulated in schizophrenia.^[10,11]^ However, the association between *RGS4* and the risk of schizophrenia remains controversial.^[12–15]^

Meta-analysis is a useful tool for the detection of disease–gene relationships.^[16]^ In the Chinese Han population, 1 meta-analysis showed no association between the *RGS4* gene and the risk of schizophrenia^[15]^; however, in another meta-analysis, the SNP, rs951436, was found to be associated with the risk of schizophrenia.^[17]^ Therefore, the association between *RGS4* and the risk of schizophrenia remains complicated and controversial.^[17–19]^ Additional articles have since been published; thus, an updated meta-analysis is needed. Here, we conducted an updated meta-analysis to detect the association between *RGS4* gene polymorphisms and the risk of schizophrenia.

## 2 Materials and methods

### 2.1 Literature search

The systematic review and meta-analysis were conducted in adherence to the Preferred Reporting Items for Systematic Reviews and Meta-Analyses guidelines.^[20]^ A search was performed in English (PubMed, SZGene) and Chinese (CNKI, Wanfang, and Weipu) databases with the following keywords: “the regulator of G-protein signaling 4” or “*RGS4*”, and “schizophrenia”. References to related articles were also reviewed for further data.

### 2.2 Identification and eligibility of relevant studies

The inclusion criteria were: studies with a case–control design; involvement of patients with schizophrenia; available allele or genotype frequencies; and published before May 12, 2020. The authors were emailed if there was no genotype frequency mentioned in the article. The exclusion criteria were: family-based studies; no control group data; no detailed genotype frequency data after emailing the authors; and duplicate samples.^[21]^ Information regarding the author, year, country, ethnicity, controls source, mean age of the control group, number of samples, diagnostic criteria, gender index the of cases and controls, and genotypes of the cases and controls were collected.

### 2.3 Statistical analysis

The meta-analysis was conducted using Stata version 10.0 (Stata Corp., College Station, TX). In the control group, the *P* value of Hardy–Weinberg equilibrium was calculated. Parameters, such as the odds ratios (ORs), 95% confidence intervals (CIs), and *P* values of association (*P*_*z*_), were calculated to detect the association in 5 genetic models,^[22]^ using the random effects model.^[21,23]^ The heterogeneity of the studies (*P*_*h*_) was determined by Cochran chi-square-based Q-statistic test. To assess the heterogeneity, subgroup analyses by ethnicity and control source were performed.^[24]^ The studies were classified by control source into community-based (participants from the general population) and hospital-based (participants from a hospital) groups.^[25]^ The Egger test was conducted to detect the publication bias, which could be visualized using a funnel plot. To assess the impact of each study on the pooled results, sensitivity analysis was performed by removing single studies in turn. The power was calculated using the PS program.^[26]^ The threshold for statistical significance was *P* < .05 in all tests.

## 3 Results

### 3.1 Description of studies

**Figure 1:**
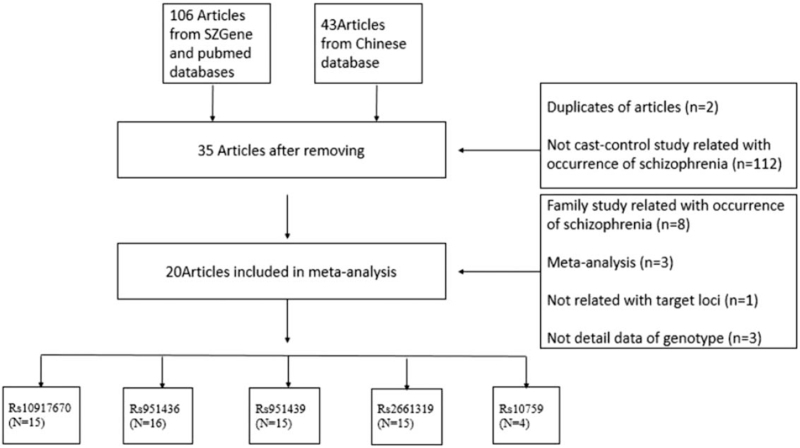
Article selection process in the present meta-analysis.

A total of 259 English and 46 Chinese articles were found, with 20 articles being eligible for analysis following exclusion (Fig. 1). The data regarding the genotypes in articles^[11,14,27]^ were unavailable. Date in 8 articles^[15,18,28–33]^ were analyzed in previous meta-analyses,^[17–19]^ however, data in the other 12 articles were not included in previous meta-analyses. Table 1 described the detailed characteristics of the 20 eligible studies. There were 15 articles regarding rs10917670,^[15,18,28–32,34–41]^ 16 regarding rs951436,^[12,13,15,18,28–34,36,38,39,42]^ 15 regarding rs951439,^[15,18,28–34,36,38–41,43]^ 15 regarding rs2661319^[15,18,28–40]^ and 4 regarding rs10759.^[13,38,39,41]^ There were less than 4 articles regarding other SNPs of the *RGS4* gene; therefore, these were not included in the present meta-analysis. The SNPs rs10917670, rs951436, and rs951439, are located in the promoter region, rs2661319 is located in the first intron, and rs10759 is located in the 3’ untranslated region.

**Table 1:** Baseline characteristics of eligible studies in the present meta-analysis.

Author	Year	Country	Ethnicity	Controls source	Mean age of control group	Diagnostic criteria	Gender index (case)	Gender index (control)
Réthelyi	2010	Hungarian	Caucasian	Community-based	39.9 ± 15.0	DSM-IV	1.174	1.381
Jönsson	2012	Scandinavian	Caucasian	Community-based	44.1 ± 11.8	DSM-III	0.712	0.736
So	2008	China	East Asia	Hospital-based	41.9 ± 9.79	DSM-IV	0.404	0.691
Guo	2006	China	East Asia	Community-based	25.87 ± 7.58	DSM-IV	0.767	0.811
Kampman	2006	Finland	Caucasian	Community-based	44.5 ± 11.1	DSM-IV	0.711	0.852
Rizig	2006	UK	Caucasian	Community-based		ICD10		
Zhang	2005	UK	Caucasian	Community-based		DSM-IV	0.389	0.754
Sobell	2005	USA	Caucasian	Hospital-based	66.2 ± 10.6	DSM-III-R		
Cordeiro	2005	Brazil	Caucasian	Community-based		DSM-IV		
Prasad	2005	USA	Caucasian	Community-based	24.74 ± 7.23	DSM-IV	0.429	0.929
Morris	2004	Irish	Caucasian	Community-based		DSM-IIIR		
Williams	2004	UK	Caucasian	Community-based	44.93 ± 12.04	DSM-IV	0.468	0.488
Bakker	2007	Dutch	Caucasian	Community-based		DSM-IV		
Betcheva	2009	Bulgaria	Caucasian	Community-based	50.5 ± 16.0	DSM-IV	1.041	0.923
Chowdari	2002	USA	Caucasian	Community-based		DSM-IV		
Sanders	2008	USA, Australia	Caucasian	Community-based		DSM-IV	0.441	
Wood	2007	US	Caucasian	Community-based		DSM-IV		
Ishiguro	2006	Japan	East Asia	Community-based	49.0 ± 14.3	DSM-IV	0.818	0.882
Yue	2007	China	East Asia	Community-based	30 ± 8	ICD-10	0.92	0.857
Qian	2005	China	East Asia	Community-based	30.8 ± 15.78	DSM-IIIR	0.936	0.79

DSM-IV = Diagnostic and Statistical Manual– Fourth Edition.

### 3.2 Results of data analysis

#### 3.2.1 There is no association between rs10917670 and the risk of schizophrenia

**Figure 2:**
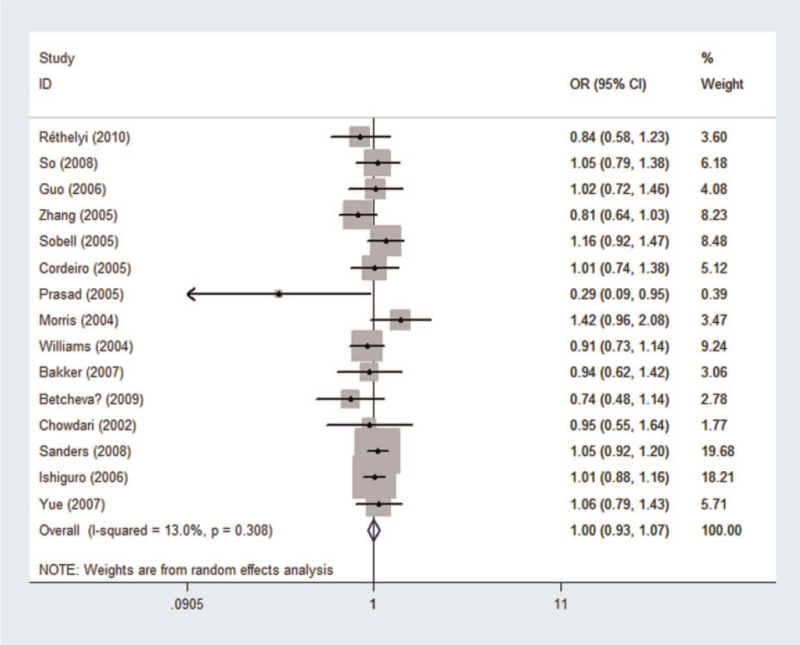
Forest plot of the association between rs10917670 and schizophrenia using a recessive model (GG vs GA + AA). CI = confidence interval, OR = odds ratio.

Genotype frequency of 8046 cases and 8837 controls was used to perform pooled and subgroup analyses using the random effects model (see Table S1, Supplemental Digital Content, which illustrated genotype distribution and allele frequency of rs10917670). Results of the pooled and subgroup analyses were summarized in Tables 2 and 3. Using the recessive model (Fig. 2), no association was found between rs10917670 and the risk of schizophrenia in the pooled analysis (*P*_*z*_ = .946, OR = 0.997, 95% CI = 0.926-1.074). No association was detected in the subgroup analyses by ethnicity or control source. Moreover, no significant heterogeneity was observed in the pooled or subgroup analyses.

**Table 2:** Pooled association of *RGS4* polymorphisms with schizophrenia.

Loci	Genetic model	Studies (n)	Statistical	OR	95% CI	*P*_z_	*I* ^2^	*P*_h_	*P*_e_
rs10917670	Allele contrast	15	Random	1.011	0.929-1.052	.72	39.40	.058	.553
	Homozygous codominant	15	Random	1.022	0.906-1.153	.725	33	.104	.663
	Heterozygous codominant	15	Random	1.048	0.954-1.150	.332	13.3	.304	.514
	Dominant	15	Random	1.045	0.944-1.157	.393	29.4	.136	.932
	Recessive	15	Random	0.997	0.926-1.074	.946	13	.308	.198
rs951436	Allele contrast	16	Random	1.039	0.967-1.116	.298	61.5	.001	.413
	Homozygous codominant	16	Random	0.971	0.852-1.107	.664	53.2	.006	.795
	Heterozygous codominant	16	Random	1.012	0.943-1.086	.741	0	.601	.86
	Dominant	16	Random	0.998	0.918-1.085	.964	26.4	.158	.931
	Recessive	16	Random	0.965	0.870-1.072	.51	52.5	.007	.619
rs951439	Allele contrast	15	Random	1.031	0.890-1.054	.461	69.6	0	.276
	Homozygous codominant	14	Random	1.018	0.886-1.170	.803	47.7	.024	.229
	Heterozygous codominant	14	Random	1.036	0.952-1.127	.416	0	.944	.674
	Dominant	14	Random	1.036	0.952-1.128	.414	6.1	.385	.324
	Recessive	14	Random	0.998	0.905-1.100	.969	44.3	.038	.139
rs2661319	Allele contrast	15	Random	1.068	1.009-1.130	.023	32.4	.109	.125
	Homozygous codominant	15	Random	1.126	1.009-1.256	.034	27.2	.156	.211
	Heterozygous codominant	15	Random	1.066	0.992-1.145	.082	0	.681	.016
	Dominant	15	Random	1.087	1.016-1.164	.016	0	.513	.027
	Recessive	15	Random	1.101	1.002-1.211	.046	34.9	.09	.424
rs10759	Allele contrast	4	Random	1.148	0.728-0.997	.046	59.2	.062	.786
	Homozygous codominant	4	Random	1.427	0.969-2.101	.072	63.2	.043	.742
	Heterozygous codominant	4	Random	1.133	0.952-1.350	.161	0	.865	.4
	Dominant	4	Random	1.226	1.038-1.448	.016	0	.516	.431
	Recessive	4	Random	1.254	0.974-1.615	.079	67.1	.028	.947

ORs = odds ratios, *P*_*e*_* = P* values of publication bias, *P*_*h*_* = P* values of heterogeneity, *P*_*z*_* = P* values of association, RGS4 = regulator of G-protein signaling 4.

**Table 3:** Subgroup association of *RGS4* polymorphisms with schizophrenia.

Loci	Subgroup analysis	Studies (n)	OR	95% CI	*P*_*z*_	*I* ^2^	*P*_*h*_
rs10917670	Caucasians	11	0.971	0.865-1.090	.618	36.5	.107
	East Asia	4	1.023	0.916-1.142	.685	0	.988
	Population-based	13	0.978	0.900-1.062	.59	15.5	.288
	Hospital-based	2	1.114	0.931-1.334	.238	0	.562
rs951436	Caucasians	13	1.017	0.905-1.144	.772	48.2	.026
	East Asia	3	0.811	0.666-0.987	.036	40	.189
	Population-based	14	0.997	0.892-1.114	.955	52.1	.012
	Hospital-based	2	0.789	0.643-0.968	.023	0	.547
rs951439	Caucasians	10	1	0.875-1.142	.999	28.3	.184
	East Asia	4	1.084	0.954-1.233	.216	0	.898
	Population-based	12	1.013	0.919-1.116	.796	11.2	.335
	Hospital-based	2	1.164	0.937-1.445	.17	0	.625
rs2661319	Caucasians	12	1.059	0.965-1.162	.229	10.4	.343
	East Asia	3	1.13	1.009-1.266	.035	0	.906
	Population-based	13	1.073	0.997-1.155	.061	1.9	.427
	Hospital-based	2	1.192	0.974-1.458	.089	0	.838
rs10759	Caucasians	3	1.132	0.928–1.380	.221	0	.917
	East Asia	1	1.482	1.092-2.011	.012	–	–

ORs = odds ratios, *P*_*h*_* = P* values of heterogeneity, *P*_*z*_* = P* values of association, RGS4 = regulator of G-protein signaling 4.

#### 3.2.2 There was an association between rs951436 and the risk of schizophrenia in the East Asian and hospital-based subgroup analyses

**Figure 3:**
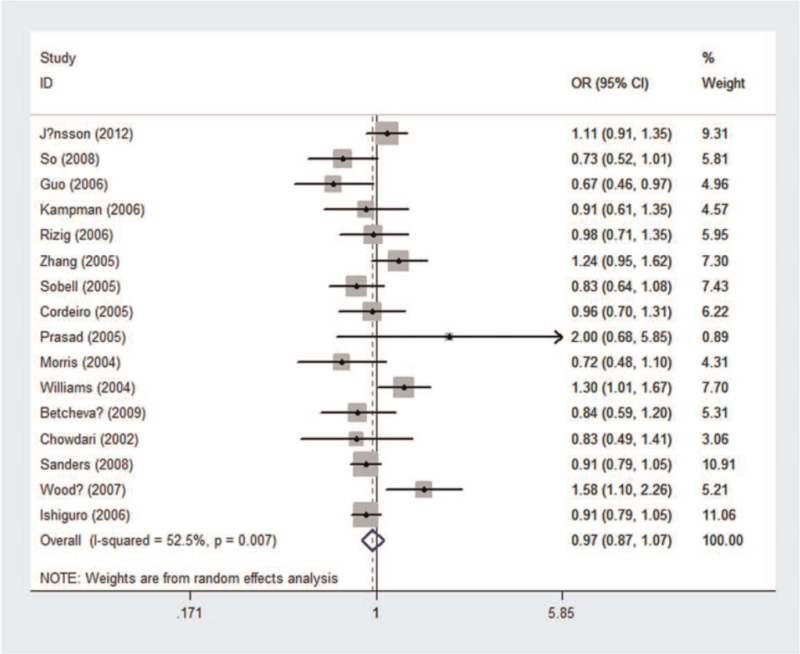
Forest plot of the association between rs951436 and schizophrenia using a recessive model (TT vs TG + GG). CI = confidence interval, OR = odds ratio.

Pooled and subgroup analyses of 8990 cases and 10,568 controls were performed (see Table S2, Supplemental Digital Content, which illustrated genotype distribution and allele frequency of rs951436). No association was found between rs951436 and the risk of schizophrenia (*P*_*z*_ = .51, OR = 0.965, 95% CI = 0.870-1.072) using the recessive model (Fig. 3). An association was detected in the East Asian (*P*_*z*_ = .036, OR = 0.811, 95% CI = 0.666-0.987) and hospital-based (*P*_*z*_ = .023, OR = 0.789, 95% CI = 0.643-0.968) subgroup analyses. Significant heterogeneity was observed in the pooled analysis (*P*_*h*_ = .007, *I*_*2*_ = 52.5%).

#### 3.2.3 There was no association between rs951439 and the risk of schizophrenia

**Figure 4:**
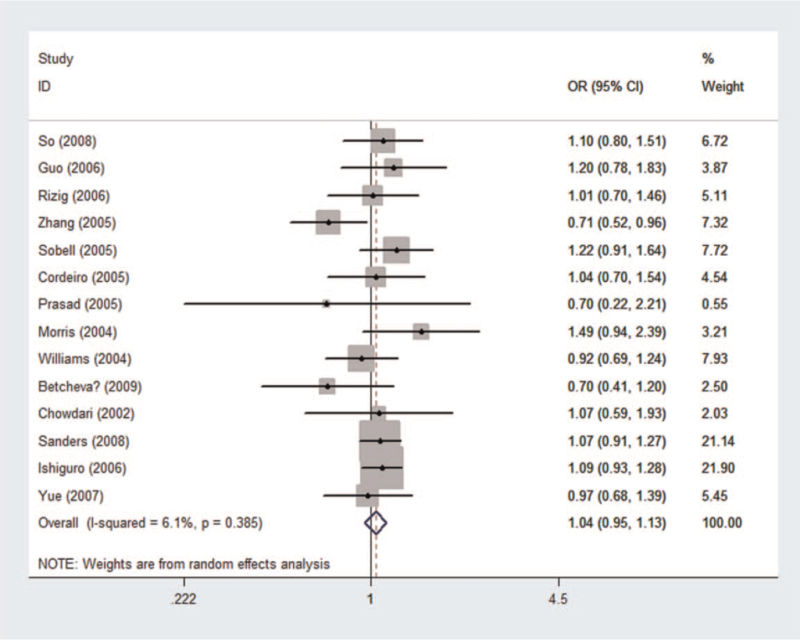
Forest plot of the association between rs951439 and schizophrenia using a dominant model (GG + GA vs AA). CI = confidence interval, OR = odds ratio.

To evaluate the relationship between rs951439 and the risk of schizophrenia, 7995 cases and 8646 controls were included in the pooled and subgroup analyses (see Table S3, Supplemental Digital Content, which illustrated genotype distribution and allele frequency of rs951439). Detailed genotype frequencies were not available in^[43]^; thus, these data were only included in the allele contrast. No relationship between rs951439 and the risk of schizophrenia was detected in the pooled analysis (*P*_*z*_ = .414, OR = 1.036, 95% CI = 0.952-1.128) using the dominant model (Fig. 4) or in the subgroup analyses by ethnicity and control source. No significant heterogeneity was observed in the pooled or subgroup analyses.

#### 3.2.4 Rs2661319 might be a risk factor for schizophrenia

**Figure 5:**
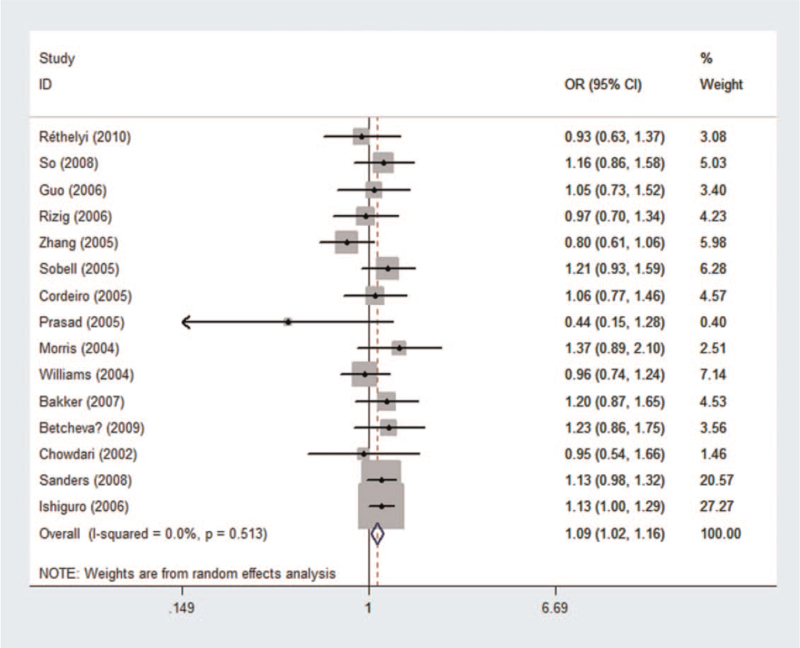
Forest plot of the association between rs2661319 and schizophrenia using a dominant model (CC + CT vs TT). CI = confidence interval, OR = odds ratio.

Pooled and subgroup analyses of 8320 cases and 9440 controls were performed (see Table S4, Supplemental Digital Content, which illustrated genotype distribution and allele frequency of rs2661319). Of the 5 genetic models, significant differences were detected when using allele contrast (C vs T, *P*_*z*_ = .023), homozygous codominant (CC vs TT, *P*_*z*_ = .034), dominant (CC + CT vs TT, *P*_*z*_ = .016), and recessive (CC vs CT + TT, *P*_*z*_ = .046). According to the dominant model (Fig. 5), the genotype CC + CT might be a risk factor for schizophrenia (*P*_*z*_ = .016, OR = 1.087, 95% CI = 1.016-1.164). An association was detected in the East Asian subgroup analysis (*P*_*z*_ = .035, OR = 1.13, 95% CI = 1.009-1.266), with a power of 0.694. No significant heterogeneity was observed in the pooled or subgroup analyses.

#### 3.2.5 Genotype CC + CA of rs10759 might be a risk factor for schizophrenia

**Figure 6:**
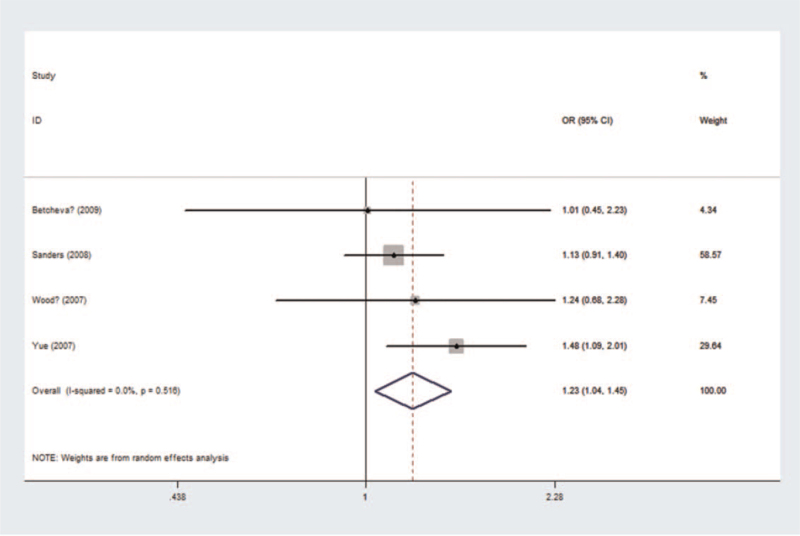
Forest plot of the association between rs10759 and schizophrenia using a dominant model (CC + CA vs AA). CI = confidence interval, OR = odds ratio.

A total of 2752 cases and 2866 controls were analyzed in pooled and subgroup analyses (see Table S5, Supplemental Digital Content, which illustrated genotype distribution and allele frequency of rs10759). Significant differences were observed in 2 of the genetic models, allele contrast (C vs A, *P*_*z*_ = .046) and dominant (CC + CA vs AA, *P*_*z*_ = .016). Using the random effects model, the dominant model was selected (Fig. 6). The genotype CC + CA of rs10759 was a risk factor for schizophrenia (*P*_*z*_ = .016, OR = 1.226, 95% CI = 1.038-1.448), with a power of 0.694. An association was found in the East Asian population (*P*_*z*_ = .012, OR = 1.482, 95% CI = 1.092-2.011). No significant heterogeneity was observed in the pooled or subgroup analyses.

#### 3.2.6 Sensitivity analysis

Sensitivity analysis was conducted by omitting each study in turn. The results showed that pooled ORs did not change significantly; thus, the results were considered stable and reasonable.

#### 3.2.7 Publication bias

Publication bias could be visualized using funnel plots. No evidence of publication bias was found in the pooled analysis (see Figures S1-S5, Supplemental Digital Content, which visualized publication bias using funnel plots for rs10917670, rs951436, rs951439, rs2661319, and rs10759, respectively).

## 4 Discussion

No association between rs10917670 and rs951439 and the risk of schizophrenia was detected in the present study, which was consistent with previous meta-analyses.^[17–19]^ In the East Asian and hospital-based subgroup analyses, an association between the genotype TT of rs951436 and the risk of schizophrenia was found; however, this relationship was not detected in the pooled analysis. Therefore, the geographical environment, culture, lifestyle, and genetic background might affect polymorphisms.^[28,31,33]^ It was studied that rs951436 was associated with magnetic resonance imaging measurements of functional activation and connectivity related to working memory, an intermediate phenotype of schizophrenia.^[44]^ Moreover, Prasad et al^[36]^ reported that rs951436 was related the volume of dorsolateral prefrontal cortex (DLPFC). But the mechanism remained unclear.

Rs2661319 and rs10759 were found to be associated with the risk of schizophrenia in the present study, which was inconsistent with previous meta-analyses. It was detected by subgroup analyses that the East Asian population contributed to this association. It was previously reported that rs2661319 was related to *RGS4*-1 mRNA level, which was decreased in the postmortem DLPFC of schizophrenic patients.^[11]^ Moreover, rs2661319 was demonstrated to be associated with a more severe baseline total PANSS score and the treatment effect of perphenazine.^[45]^ The rs10759 polymorphism was suggested to increase the risk of schizophrenia by altering the binding of miRNA-124 to its target.^[46]^ MiRNA-124 might bind to the 3′UTR of mRNAs containing target sites, resulting in miRNA-mediated gene silencing, translational inhibition, and induction of mRNA de-adenylation or decay.^[47]^ The level of RGS4 might be decreased, leading to dysfunction of neurotransmission.

More relevant data were included in our meta-analysis than those in previous meta-analyses, for instance, an increased number of more SNPs (5), and databases ((PubMed and SZGene, CNKI, Wanfang, and Weipu). However, the results described herein should be interpreted with caution. First, in the present study, the East Asian population contributed to the association between the *RGS4* gene and the risk of schizophrenia; however, the sample size was relatively small, and the power was low. Further articles are needed to form a representative and comprehensive conclusion. Second, family-based and functional studies were not included in the present meta-analysis. In addition, it was reported that there was an association between DLPFC volume and *RGS4* genotype interacting with *COMT* rs4818^[48]^; thus, this association warrants further gene–gene interaction^[49,50]^ and functional studies.

## 5 Conclusion

No association between rs10917670 and the risk of schizophrenia was found. In the East Asian and hospital-based subgroup analyses, an association between rs951436 and the risk of schizophrenia was demonstrated. No association between rs951439 and the risk of schizophrenia was detected. The genotypes CC + CT of rs2661319 and CC + CA of rs10759 might be risk factors for schizophrenia, and the East Asian population contributed to this association. Further updated gene–gene interaction and functional studies are needed.

## Acknowledgments

Feng-Ling Xu, Jun Yao, and Bao-Jie Wang were worthy of acknowledgments.

## Author contributions

BW designed the study and wrote the protocol. FX managed the literature search. FX performed analyses. The manuscript was written by FX, and corrected by JY.

**Conceptualization:** Feng-Ling Xu, Bao-Jie Wang.

**Data curation:** Feng-Ling Xu.

**Formal analysis:** Feng-Ling Xu.

**Investigation:** Feng-Ling Xu.

**Methodology:** Bao-Jie Wang.

**Project administration:** Feng-Ling Xu.

**Software:** Feng-Ling Xu.

**Supervision:** Jun Yao, Bao-Jie Wang.

**Validation:** Bao-Jie Wang.

**Visualization:** Bao-Jie Wang.

**Writing – original draft:** Feng-Ling Xu.

**Writing – review & editing:** Jun Yao.

## Supplementary Material

Supplemental Digital Content

## Supplementary Material

Supplemental Digital Content

## Supplementary Material

Supplemental Digital Content

## Supplementary Material

Supplemental Digital Content

## Supplementary Material

Supplemental Digital Content

## Supplementary Material

Supplemental Digital Content

## Supplementary Material

Supplemental Digital Content

## Supplementary Material

Supplemental Digital Content

## Supplementary Material

Supplemental Digital Content

## Supplementary Material

Supplemental Digital Content
