# Supplementary material for: Association between RGS4 gene polymorphisms and schizophrenia: A protocol for systematic review and meta-analysis
Source: Medicine (Baltimore). 2021 Nov 5;100(44):e27607. doi: 10.1097/MD.0000000000027607 (PMC8568470; doi:10.1097/MD.0000000000027607)
Supplement: Supplemental Digital Content [file medi-100-e27607-s001.docx]

Supplemental Digital Content (Table S1). Genotype distribution and allele frequency of rs10917670

| Author | Year | Genotype distribution | | | | | | |  | Allele frequency | | | | |
| --- | --- | --- | --- | --- | --- | --- | --- | --- | --- | --- | --- | --- | --- | --- |
|  |  | Case, n | | |  | Control, n | | |  | Case, n | |  | Control, n | |
|  |  | AA | AG | GG |  | AA | AG | GG | *P*_HWE_ | A | G |  | A | G |
| Réthelyi | 2010 | 60 | 139 | 81 |  | 42 | 113 | 75 | 0.961 | 259 | 301 |  | 197 | 263 |
| So | 2008 | 94 | 216 | 132 |  | 107 | 249 | 145 | 0.996 | 404 | 480 |  | 461 | 541 |
| Guo | 2006 | 49 | 146 | 91 |  | 55 | 140 | 89 | 0.997 | 244 | 328 |  | 250 | 318 |
| Zhang | 2005 | 107 | 284 | 189 |  | 94 | 295 | 231 | 0.991 | 498 | 662 |  | 483 | 757 |
| Sobell | 2005 | 90 | 273 | 205 |  | 129 | 335 | 225 | 0.827 | 453 | 683 |  | 593 | 785 |
| [Cordeiro](https://www.ncbi.nlm.nih.gov/pubmed/?term=Cordeiro Q[Author]&cauthor=true&cauthor_uid=15660667) | 2005 | 45 | 140 | 85 |  | 101 | 293 | 179 | 0.315 | 230 | 310 |  | 495 | 651 |
| Prasad | 2005 | 9 | 13 | 6 |  | 7 | 7 | 13 | 0.018 | 31 | 25 |  | 21 | 33 |
| Morris | 2004 | 40 | 119 | 90 |  | 50 | 115 | 66 | 0.994 | 199 | 299 |  | 215 | 247 |
| Williams | 2004 | 116 | 338 | 231 |  | 114 | 330 | 247 | 0.831 | 570 | 800 |  | 558 | 824 |
| Bakker | 2007 | 82 | 135 | 40 |  | 205 | 214 | 82 | 0.042 | 299 | 215 |  | 624 | 378 |
| Betcheva | 2009 | 37 | 92 | 56 |  | 28 | 88 | 68 | 0.957 | 166 | 204 |  | 144 | 224 |
| Chowdari | 2002 | 31 | 69 | 46 |  | 21 | 45 | 32 | 0.489 | 131 | 161 |  | 87 | 109 |
| Sanders | 2008 | 317 | 906 | 647 |  | 357 | 977 | 669 | 0.993 | 1540 | 2200 |  | 1691 | 2315 |
| Ishiguro | 2006 | 392 | 866 | 556 |  | 367 | 895 | 553 | 0.888 | 1650 | 1978 |  | 1629 | 2001 |
| Yue | 2007 | 49 | 196 | 141 |  | 68 | 185 | 137 | 0.684 | 294 | 478 |  | 324 | 459 |
