# Supplementary material for: Association between RGS4 gene polymorphisms and schizophrenia: A protocol for systematic review and meta-analysis
Source: Medicine (Baltimore). 2021 Nov 5;100(44):e27607. doi: 10.1097/MD.0000000000027607 (PMC8568470; doi:10.1097/MD.0000000000027607)
Supplement: Supplemental Digital Content [file medi-100-e27607-s003.docx]

Supplemental Digital Content (Table S2). Genotype distribution and allele frequency of rs951436

| Author | Year | Genotype distribution | | | | | | |  | Allele frequency | | | | |
| --- | --- | --- | --- | --- | --- | --- | --- | --- | --- | --- | --- | --- | --- | --- |
|  |  | Cases, n | | |  | Controls, n | | |  | Cases, % | |  | Controls, % | |
|  |  | TT | TG | GG |  | TT | TG | GG | *P*_HWE_ | T | G |  | T | G |
| Jönsson | 2012 | 223 | 416 | 197 |  | 349 | 711 | 352 | 0.790 | 862 | 810 |  | 1409 | 1415 |
| So | 2008 | 88 | 201 | 85 |  | 106 | 178 | 73 | 0.913 | 377 | 371 |  | 390 | 324 |
| Guo | 2006 | 65 | 141 | 71 |  | 88 | 125 | 67 | 0.088 | 283 | 271 |  | 259 | 301 |
| Kampman | 2006 | 47 | 114 | 58 |  | 90 | 190 | 109 | 0.682 | 155 | 169 |  | 370 | 408 |
| Rizig | 2006 | 92 | 202 | 112 |  | 99 | 214 | 116 | 0.987 | 386 | 426 |  | 412 | 446 |
| Zhang | 2005 | 151 | 290 | 139 |  | 137 | 309 | 174 | 0.993 | 592 | 568 |  | 583 | 657 |
| Sobell | 2005 | 128 | 300 | 140 |  | 179 | 340 | 170 | 0.735 | 556 | 580 |  | 698 | 680 |
| [Cordeiro](https://www.ncbi.nlm.nih.gov/pubmed/?term=Cordeiro Q[Author]&cauthor=true&cauthor_uid=15660667) | 2005 | 86 | 142 | 40 |  | 188 | 299 | 83 | 0.040 | 314 | 222 |  | 675 | 465 |
| Prasad | 2005 | 15 | 11 | 4 |  | 9 | 11 | 7 | 0.348 | 41 | 19 |  | 29 | 25 |
| Morris | 2004 | 54 | 124 | 71 |  | 64 | 115 | 52 | 0.980 | 232 | 266 |  | 243 | 219 |
| Williams | 2004 | 180 | 342 | 161 |  | 146 | 340 | 189 | 0.765 | 702 | 664 |  | 632 | 718 |
| Betcheva | 2009 | 55 | 113 | 87 |  | 136 | 277 | 139 | 0.932 | 388 | 287 |  | 549 | 555 |
| Chowdari | 2002 | 39 | 75 | 35 |  | 38 | 59 | 30 | 0.449 | 153 | 145 |  | 135 | 119 |
| Sanders | 2008 | 458 | 935 | 477 |  | 527 | 1000 | 475 | 0.988 | 1851 | 1889 |  | 2054 | 1950 |
| Wood | 2007 | 101 | 137 | 72 |  | 68 | 148 | 74 | 0.719 | 339 | 281 |  | 284 | 296 |
| Ishiguro | 2006 | 529 | 978 | 409 |  | 566 | 932 | 420 | 0.325 | 2036 | 1796 |  | 2064 | 1772 |
