# Supplementary material for: Association between RGS4 gene polymorphisms and schizophrenia: A protocol for systematic review and meta-analysis
Source: Medicine (Baltimore). 2021 Nov 5;100(44):e27607. doi: 10.1097/MD.0000000000027607 (PMC8568470; doi:10.1097/MD.0000000000027607)
Supplement: Supplemental Digital Content [file medi-100-e27607-s004.docx]

Supplemental Digital Content (Table S3). Genotype distribution and allele frequency of rs951439

| Author | Year | Genotype distribution | | | | | | |  | Allele frequency | | | | |
| --- | --- | --- | --- | --- | --- | --- | --- | --- | --- | --- | --- | --- | --- | --- |
|  |  | Cases, n | | |  | Controls, n | | |  | Cases, % | |  | Controls, % | |
|  |  | AA | AG | GG |  | AA | AG | GG | *P*_HWE_ | A | G |  | A | G |
| So | 2008 | 84 | 206 | 125 |  | 112 | 254 | 148 | 0.877 | 374 | 456 |  | 478 | 550 |
| Guo | 2006 | 49 | 145 | 92 |  | 56 | 143 | 83 | 0.692 | 243 | 329 |  | 255 | 309 |
| Rizig | 2006 | 66 | 196 | 147 |  | 70 | 206 | 153 | 0.962 | 328 | 490 |  | 346 | 512 |
| Zhang | 2005 | 112 | 286 | 182 |  | 90 | 292 | 238 | 0.977 | 510 | 650 |  | 472 | 768 |
| Sobell | 2005 | 90 | 273 | 205 |  | 129 | 335 | 225 | 0.827 | 453 | 683 |  | 593 | 785 |
| [Cordeiro](https://www.ncbi.nlm.nih.gov/pubmed/?term=Cordeiro Q[Author]&cauthor=true&cauthor_uid=15660667) | 2005 | 44 | 136 | 83 |  | 97 | 292 | 172 | 0.156 | 224 | 302 |  | 486 | 636 |
| Prasad | 2005 | 10 | 13 | 7 |  | 7 | 7 | 13 | 0.018 | 33 | 27 |  | 21 | 33 |
| Morris | 2004 | 38 | 119 | 92 |  | 49 | 115 | 67 | 0.979 | 195 | 303 |  | 213 | 249 |
| Williams | 2004 | 111 | 344 | 231 |  | 104 | 333 | 250 | 0.689 | 566 | 806 |  | 541 | 833 |
| Betcheva | 2009 | 39 | 92 | 54 |  | 29 | 88 | 66 | 0.970 | 170 | 200 |  | 146 | 220 |
| Chowdari | 2002 | 29 | 74 | 46 |  | 26 | 62 | 39 | 0.881 | 132 | 166 |  | 114 | 140 |
| Sanders | 2008 | 313 | 904 | 653 |  | 355 | 976 | 671 | 0.998 | 1530 | 2210 |  | 1686 | 2318 |
| Ishiguro | 2006 | 342 | 956 | 621 |  | 364 | 967 | 573 | 0.219 | 1640 | 2198 |  | 1695 | 2113 |
| Yue | 2007 | 76 | 195 | 115 |  | 75 | 183 | 132 | 0.418 | 347 | 425 |  | 333 | 447 |
| Qian | 2005 | - | - | - |  | - | - | - | - | 498 | 598 |  | 613 | 493 |
