# Supplementary material for: Association between RGS4 gene polymorphisms and schizophrenia: A protocol for systematic review and meta-analysis
Source: Medicine (Baltimore). 2021 Nov 5;100(44):e27607. doi: 10.1097/MD.0000000000027607 (PMC8568470; doi:10.1097/MD.0000000000027607)
Supplement: Supplemental Digital Content [file medi-100-e27607-s005.docx]

Supplemental Digital Content (Table S4). Genotype distribution and allele frequency of rs2661319

| Author | Year | Genotype distribution | | | | | | |  | Allele frequency | | | | |
| --- | --- | --- | --- | --- | --- | --- | --- | --- | --- | --- | --- | --- | --- | --- |
|  |  | Cases, n | | |  | Controls, n | | |  | Cases, % | |  | Controls, % | |
|  |  | CC | CT | TT |  | CC | CT | TT | *P*_HWE_ | C | T |  | C | T |
| Réthelyi | 2010 | 59 | 139 | 82 |  | 51 | 115 | 64 | 0.961 | 257 | 303 |  | 217 | 243 |
| So | 2008 | 123 | 207 | 95 |  | 121 | 267 | 130 | 0.478 | 453 | 397 |  | 509 | 527 |
| Guo | 2006 | 68 | 134 | 79 |  | 65 | 130 | 80 | 0.391 | 270 | 292 |  | 260 | 290 |
| Rizig | 2006 | 120 | 207 | 89 |  | 128 | 214 | 90 | 0.975 | 447 | 385 |  | 470 | 394 |
| Zhang | 2005 | 157 | 290 | 134 |  | 194 | 306 | 120 | 0.973 | 604 | 558 |  | 694 | 546 |
| Sobell | 2005 | 155 | 299 | 114 |  | 193 | 335 | 161 | 0.504 | 609 | 527 |  | 721 | 657 |
| [Cordeiro](https://www.ncbi.nlm.nih.gov/pubmed/?term=Cordeiro Q[Author]&cauthor=true&cauthor_uid=15660667) | 2005 | 49 | 139 | 79 |  | 89 | 305 | 176 | 0.022 | 237 | 297 |  | 483 | 657 |
| Prasad | 2005 | 4 | 10 | 16 |  | 7 | 11 | 9 | 0.348 | 18 | 42 |  | 25 | 29 |
| Morris | 2004 | 76 | 123 | 50 |  | 57 | 115 | 59 | 0.948 | 275 | 223 |  | 229 | 233 |
| Williams | 2004 | 277 | 331 | 165 |  | 205 | 329 | 139 | 0.740 | 885 | 661 |  | 739 | 607 |
| Bakker | 2007 | 62 | 136 | 75 |  | 113 | 286 | 181 | 0.999 | 262 | 284 |  | 510 | 650 |
| Betcheva | 2009 | 78 | 114 | 53 |  | 124 | 290 | 140 | 0.261 | 270 | 220 |  | 538 | 570 |
| Chowdari | 2002 | 39 | 73 | 35 |  | 30 | 68 | 29 | 0.424 | 151 | 143 |  | 128 | 126 |
| Sanders | 2008 | 525 | 932 | 413 |  | 515 | 1001 | 487 | 0.989 | 1982 | 1758 |  | 2031 | 1975 |
| Ishiguro | 2006 | 264 | 929 | 722 |  | 267 | 866 | 778 | 0.297 | 1457 | 2373 |  | 1400 | 2422 |
