# Supplementary material for: Association between RGS4 gene polymorphisms and schizophrenia: A protocol for systematic review and meta-analysis
Source: Medicine (Baltimore). 2021 Nov 5;100(44):e27607. doi: 10.1097/MD.0000000000027607 (PMC8568470; doi:10.1097/MD.0000000000027607)
Supplement: Supplemental Digital Content [file medi-100-e27607-s006.docx]

Supplemental Digital Content (Table S5). Genotype distribution and allele frequency of rs10759

| Author | Year | Genotype distribution | | | | | | |  | Allele frequency | | | | |
| --- | --- | --- | --- | --- | --- | --- | --- | --- | --- | --- | --- | --- | --- | --- |
|  |  | Cases, n | | |  | Controls, n | | |  | Cases, % | |  | Controls, % | |
|  |  | AA | AC | CC |  | AA | AC | CC | *P*_HWE_ | A | C |  | A | C |
| Betcheva | 2009 | 13 | 71 | 101 |  | 13 | 73 | 98 | 0.906 | 97 | 273 |  | 99 | 269 |
| Sanders | 2008 | 165 | 781 | 924 |  | 197 | 862 | 942 | 0.992 | 1111 | 2629 |  | 1256 | 2746 |
| Wood | 2007 | 21 | 135 | 155 |  | 24 | 133 | 134 | 0.257 | 177 | 445 |  | 181 | 401 |
| Yue | 2007 | 105 | 190 | 91 |  | 139 | 199 | 52 | 0.144 | 400 | 372 |  | 477 | 303 |
