# Supplementary material for: Association between RGS4 gene polymorphisms and schizophrenia: A protocol for systematic review and meta-analysis
Source: Medicine (Baltimore). 2021 Nov 5;100(44):e27607. doi: 10.1097/MD.0000000000027607 (PMC8568470; doi:10.1097/MD.0000000000027607)
Supplement: Supplemental Digital Content [file medi-100-e27607-s007.docx]

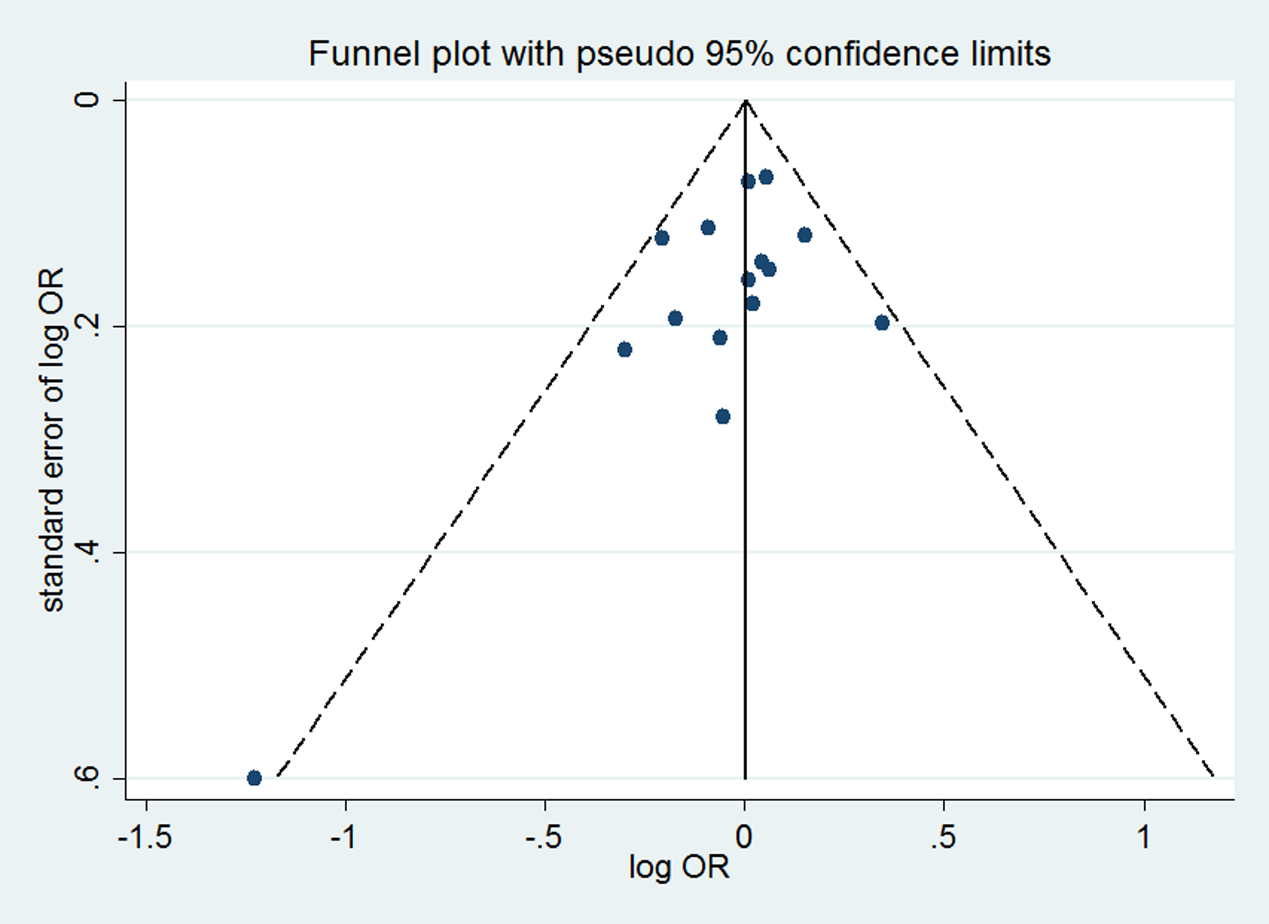


Supplemental Digital Content (Figure S1). Funnel plot analysis for the detection of publication bias in the association between the rs10917670 and schizophrenia.
